# Use of a poll‐mounted accelerometer for quantification and characterisation of equine trigeminal‐mediated headshaking

**DOI:** 10.1111/evj.14132

**Published:** 2024-07-17

**Authors:** Kirstie Jane Pickles, David J. Marlin, Jane Michelle Williams, Veronica L. H. Roberts

**Affiliations:** ^1^ School of Veterinary Medicine and Science University of Nottingham Sutton Bonington UK; ^2^ Animalweb Ltd Cambridge UK; ^3^ Hartpury University Gloucester UK; ^4^ Bristol Vet School University of Bristol Langford UK; ^5^ Present address: KP Consulting Duffield UK; ^6^ Present address: Harper Keele Veterinary School Keele Staffordshire UK

**Keywords:** accelerometer, headshaking, horse, severity, trigeminal‐mediated

## Abstract

**Background:**

Equine trigeminal‐mediated (TGM) headshaking (HS) is a neuropathic facial pain syndrome characterised by varying intensity and frequencies of head movements and signs of nasal irritation. An accurate method for quantification and/or characterisation of HS severity is lacking.

**Objectives:**

To develop and validate an objective measure of TGMHS.

**Study design:**

Prospective case control study.

**Methods:**

Horses presenting for investigation of HS were recruited alongside those presenting for forelimb lameness (LAME) and pre‐purchase examination as well as healthy controls (CONTROL). Head movement data were collected for 5 min whilst trotting on the lunge using a tri‐axial accelerometer, with a range of ±16 g and sampling rate of 800 Hz, attached to the bridle headpiece. Recordings were exported for processing. Peak detection was performed using minimum and maximum thresholds of −1 g and +1 g (corrected for gravity) and a minimum peak width of 10 samples.

**Results:**

Fifty‐six horses were included in the study; 18 TGMHS, 10 non‐TGMHS, 12 LAME and 16 CONTROL. Characteristics and frequency of vertical (*Z* axis) head movements of TGMHS horses differed significantly from other horses. TGMHS horses had peaks with greater mean and maximum positive g‐force (*P* < 0.005) and lower mean and minimum negative g‐force (*P* < 0.001), greater frequency of peaks/min (*P* < 0.001) and over 12 times greater percentage of peaks >+2 g compared with other horses (*P* < 0.001). Receiver operator curve characteristics of percentage of peaks >+2 g (CI 0.72–0.95), percentage of peaks <−2 g (CI 0.66–0.92) and percentage of peaks <−2 g and >+2 g (CI 0.72–0.96) showed excellent discrimination of TGMHS horses from LAME, CONTROL and non‐TGMHS horses.

**Main limitations:**

Referral population of horses, small sample size and control horses were not evaluated for orthopaedic disease.

**Conclusions:**

Accelerometer data from trotting exercise on the lunge provides an objective measure of HS and can differentiate between TGMHS, non‐TGMHS, normal head movements and those associated with forelimb lameness. Accelerometer use may aid HS diagnosis and monitoring of management strategies.

## Resumen

**Historial:**

La sacudida de cabeza (HS) en equinos mediada por el nervio trigémino (TGM), es un síndrome de dolor facial neuropático caracterizado por movimientos de cabeza de intensidad y frecuencia variables y por síntomas de irritación nasal. No existe un método preciso para cuantificar y/o caracterizar la gravedad de HS.

**Objetivos:**

Desarrollar y validar una medida objetiva de TGMHS.

**Diseño del Estudio:**

Estudio prospectivo de casos y controles.

**Métodos:**

Caballos presentados para la investigación de HS fueron reclutados junto a aquellos presentados para cojera de mano (LAME) y examen de pre‐compra como controles saludables (CONTROL). Datos de los movimiento de la cabeza fueron recolectados durante cinco minutos mientras trotaban a la cuerda usando un acelerómetro tri‐axial, con un rango de ±16 g y frecuencia de muestreo de 800 Hz, ajustado a la testera de la cabezada. Las grabaciones se exportaron para ser procesadas. Detección de picos fue realizada usando umbrales mínimos y máximos de −1g y + 1g (corregidos para gravedad) y un muestreo mínimo de 10 picos de ancho.

**Resultados:**

Cincuenta y seis caballos fueron incluidos en el estudio; 18 TGMHS, 10 no‐TGMHS, 12 LAME y 16 CONTROL. Las características y la frecuencia de los movimientos de cabeza verticales (eje Z) de caballos TGMHS, difirieron significativamente de otros caballos. Los caballos TGMHS presentaron picos con una fuerza‐g positiva con mayor promedio y máxima (*P* < 0.005) y una fuerza‐g negativa con menor promedio y mínima (*P* < 0.001), con mayor frecuencia de picos/min (*P* < 0.001), y un porcentaje mas de doce veces mayor de picos >+2 g en comparación a los otros caballos (*P* < 0.001). Las características de las curvas del operador del receptor de los picos promedio >+2 g (CI 0.72–0.95), del porcentaje de picos <−2 g (CI 0.66–0.92), y del porcentaje de picos <−2 g y > +2 g (CI 0.72–0.96) mostraron una excelente discriminación de caballos TGMHS con respecto a caballos LAME, CONTROL y no‐TGMHS.

**Limitaciones Principales:**

Población de caballos derivados, numero de muestra pequeño, y los caballos control no fueron evaluados por enfermedades ortopédicas.

**Conclusiones:**

Los datos de acelerómetros por trote a la cuerda, dan una medida objetiva de HS y permiten diferenciar entre TGMHS, no‐TGMHS, movimientos normales de cabeza y aquellos asociados a cojera de mano. El uso de acelerómetros puede ayudar en el diagnostico de HS y monitorear las estrategias de manejo.

## 1 INTRODUCTION

Trigeminal‐mediated headshaking (TGMHS) is an idiopathic facial pain condition of the horse which can have significant welfare implications.^1^ The trigeminal nerve is sensitised in affected horses, by a yet unknown mechanism, causing a reduced threshold for activation, which is thought to result in neuropathic pain.^2^ Clinical signs include violent head flicks, which are predominantly vertical but can also be horizontal, and/or rotatory, and signs of nasal irritation such as muzzle rubbing, snorting and striking the face with a forelimb.^1^ Currently, diagnosis is made by careful observation and exclusion of other causes of the displayed behaviour, ideally including computed tomography of the head.^3^

Published scoring systems for headshaking behaviour are semi‐quantitative at best and are still open to a high degree of subjectivity.^4^, ^5^, ^6^ The intermittent nature of clinical signs, which vary with exercise and environmental and seasonal triggers, also make ascribing disease severity difficult, especially for owners who might only see their horse for a relatively short period of time per day. Likewise, interpretation of treatment efficacy can be very challenging, particularly given the considerable placebo effect (30%) evident in owner scored trials.^4^, ^7^ A validated, objective measure of HS frequency would be extremely useful for interpretation of severity and treatment efficacy and improving the ability to correctly judge treatment outcomes would have a positive effect on welfare.

TGMHS carries a poor prognosis, with published treatments lacking consistent efficacy, and in some cases, safety and practical application.^1^, ^5^ For unresponsive horses that headshake frequently at rest, welfare is compromised and euthanasia on humane ground may be appropriate. Even when euthanasia is not performed on welfare grounds, there is wastage to the equine industry with headshaking of sufficient severity to require veterinary intervention affecting 1% of the UK equine population.^8^ An objective measure of HS frequency would again be useful for evidencing such disease severity to owners and insurance companies.

Triaxial accelerometers contain a piezoelectric sensor that generates a voltage signal in response to any change in velocity experienced in three planes and produces outputs representative of three‐dimensional movement.^9^ Their use has also been validated for medical applications, including in veterinary patients, for purposes such as measurement of canine pruritis,^10^ canine behavioural states,^11^ efficacy of canine osteoarthritis drugs,^12^ play behaviours in newborn calves^13^ and lying behaviours in horses.^14^ The aim of this study was to test the utility of a head‐mounted triaxial accelerometer for objective characterisation and measurement of head movements in TGMHS, secondly, to evaluate if such head movements differed from those of normal, non‐TGMHS or lameness‐associated head movements. We hypothesised that a wearable triaxial accelerometer would differentiate TGMHS head movements from normal, exercising head movements or those associated with lameness.

## 2 MATERIALS AND METHODS

### 2.1 Animals

Horses referred for investigation of headshaking (HS) and forelimb lameness (LAME) were included, alongside those presented for pre‐purchase examination and healthy controls (CONTROL). A convenience sample based on presentation of suitable cases in the study period of 28 April 2022 to 21 July 2023 was used.

Eligible HS cases underwent detailed assessment by authors VR and KP including anamnesis, walk and trot exercise on the lunge, physical, oral and ophthalmic examination, endoscopy of the upper respiratory tract and guttural pouches, and computed tomography of the head. Horses were scored on a three‐point headshaking scale as described by Roberts et al. 2018,^15^ where 0/3 = no headshaking, 1/3 = headshaking but of insufficient severity to interfere with ridden exercise, 2/3 = headshaking of sufficient severity as to make ridden exercise impossible or unsafe and 3/3 = headshaking at rest. Horses with an identifiable cause of HS behaviour (e.g., sinusitis, dental disease, etc.) on these ancillary examinations were classified as non‐TGMHS. The remaining HS horses were classified as idiopathic TGMHS.

Horses presenting to colleagues at the same veterinary hospital, were used as LAME and CONTROL. Lameness scores out of 10 were recorded for all LAME horses by a single sports medicine clinician. If animals were lame on both forelimbs, the score of the lamest limb was recorded.

### 2.2 Accelerometer data

**FIGURE 1:**
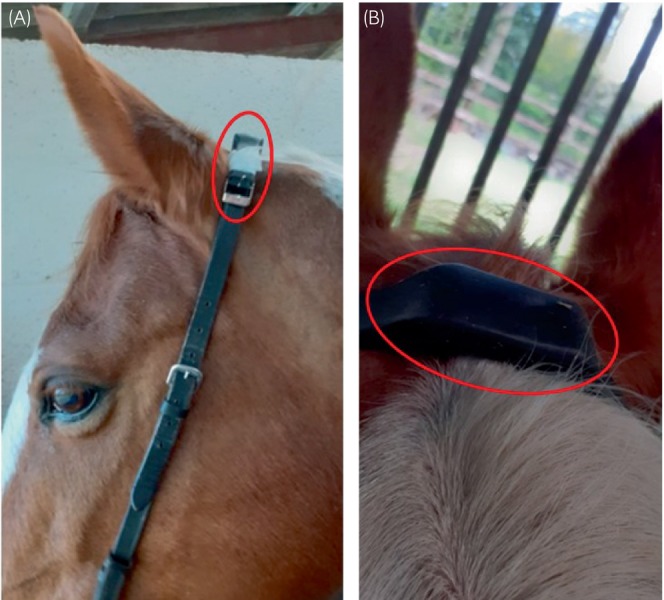
Lateral (A) and caudorostral (B) view of the position of the accelerometer fixed to the bridle headpiece. The accelerometer is shown by a red circle.

**FIGURE 2:**
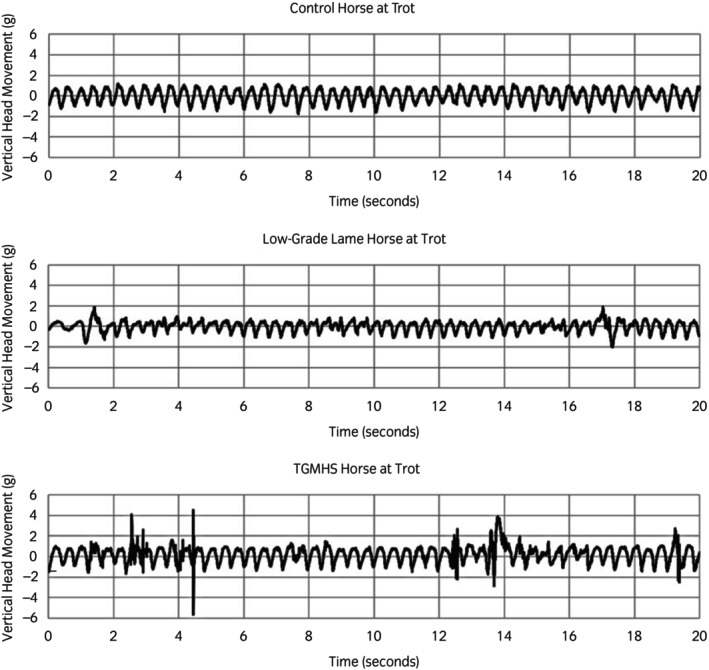
A 20‐s sample of vertical (*Z* axis) head movement data from a CONTROL horse, a LAME horse (2/10) and a horse with trigeminal‐mediated headshaking (TGMHS) using a poll‐mounted accelerometer during 5 min of lungeing. Headshaking episodes can be seen at around 2.5, 4.3, 12.5, 13.5 and 19.2 s of the TGMHS horse recording.

Head movement data were collected for 5 min whilst trotting on the lunge using a tri‐axial accelerometer (Axivity AX3, Axivity Ltd), 23.0 × 32.5 × 7.6 mm and weighing 11 g, with 13‐bit resolution, a range of ±16 g and sampling rate of 800 Hz, firmly attached to the bridle or halter headpiece (Figure 1). The sampling rate of 800 Hz was based on Fast Fourier Transform of headshaking events which showed frequency content up to 80 Hz. The Axivity AX3 has a recording capacity of 42 h at 800 Hz. In theory, whilst a lower sampling frequency according to the Nyquist sampling theorem would be acceptable, in practice sampling at 200 Hz resulted in reduction in peak heights and as a result, numbers of detected peaks, compared with 800 Hz. Accelerometer recordings were viewed in OM GUI software (Axivity Ltd), and vertical *Z* axis data (Figure 2) exported into Sigview (v 6.2.0, SignalLab e.K.) for processing. Peak detection was performed using minimum and maximum thresholds of −1 g and +1 g (corrected for gravity) and a minimum peak width of 10 samples. Filtering was not applied as visual inspection of recording did not indicate the presence of noise or interference. The detected peaks (time and actual g) were exported to Excel for further analysis. The following variables were determined: *n* peaks <1 g, *n* peaks >1 g, sum of peaks <1 g or >1 g, ratio *n* +VE peaks >1 g:*n* −VE peaks <1 g, *n* peaks <2 g, *n* peaks >2 g, sum of peaks <2 g or >2 g, mean +ve g, mean −ve g, max +ve g, min −ve g, ratio of +ve:−ve peaks, % peaks >2 g, % peaks <2 g, % peaks <2 g and % peaks >2 g, +ve peaks >1 g/min, −ve peaks <1 g/min, sum of +ve peaks >1 g and −ve peaks <1 g/min.

### 2.3 Data analysis

Analyses were performed using IBM SPSS (IBM Corp). Normality of continuous variables was tested and, as they met non‐parametric assumptions, median and IQR have been reported as measures of central tendency and non‐parametric analyses were undertaken. A series of Mann–Whitney *U* analyses assessed if difference occurred in sensor measurements (mean and maximum positive g, mean and minimum negative g, positive peaks >1 g/min, ratio of positive to negative peaks, percentage of peaks >+2 g, <−2 g and <−2 g and >+2 g) between TGMHS and all other horse groups combined. A series of Kruskal–Wallis analyses determined to identify if differences where present across the four groups (TGMHS, Non‐TGMHS, CONTROL and LAME) for all variables; where results where significant, post hoc Mann Whitney tests identified differences between groups. To reduce the risk of type I errors, adjusted alpha values were used when assessing statistical analyses. Significance was set at *P* < 0.05.

To determine if it was appropriate to include NON‐TGMHS horses in the broader ‘control’ group, ROC analysis assessed if the data could differentiate between TGMHS and NON‐TGMHS for the variables measured. Receiver operator curve characteristics for the percentage of peaks >+2 g, and percentage of peaks <−2 g and >+2 g and +VE peaks >1 g/min demonstrate that TGMHS horses can be discriminated from NON‐TGMHS horses, justifying their inclusion in the combined group of horses which were not clinically diagnosed as being a TGMHS (Table S1). Receiver‐operator characteristic (ROC) curves were plotted for individual sensor variables to assess discrimination of a horse being a TGMHS versus all other horse groups (CONTROL, LAME or non‐TGMHS) combined (1 = 100% predictability).^16^, ^17^

For ROC curves, the area under the curve (AUC) values were interpreted to assess the predictability to identify TGMHS: <0.5 suggests no discrimination; 0.7–0.8 considered acceptable; 0.8–0.9 considered excellent and >0.9 considered outstanding.^18^, ^19^ Sensitivity and specificity coordinates were examined to identify a cut‐off/threshold level for measurements, using 80% (>0.80) for both as a minimum value. These values were used to calculate positive and negative predictive values for measurements.^20^

## 3 RESULTS

### 3.1 Animals

The dataset comprised 56 adult horses: 18 TGMHS, 10 non‐TGMHS, 12 LAME and 16 CONTROL. Three CONTROL horses were presented for pre‐purchase examination with the remaining 13 being healthy controls. Of the LAME horses, 9 were lame on the right forelimb and 3 on the left forelimb. The median (range) lameness score was 2/10 (1–3). Signalment and headshaking details of TGMHS and non‐TGMHS horses are shown in Tables 1 and 2, respectively. All headshaking horses exhibited headshaking whilst being ridden and exercised on the lunge. Three of the TGMHS horses had a history of self‐mutilation, whereas none of the non‐TGMHS exhibited this behaviour. Of the TGMHS horses, 12 were graded 2/3 and 6 were graded 3/3. In the non‐TGMHS group, 6 horses were graded 2/3 and 4 horses as 3/3.

**TABLE 1:** Signalment and headshaking (HS) details of trigeminal mediated headshaking horses (*n* = 18).

Age (y)	Breed	Sex	Use	Grade/3	Self‐mutilation
12	Irish Sports Horse	G	Eventing	2	N
10	Warmblood	G	Dressage	3	N
12	German Sports Pony	G	Dressage	2	N
10	Welsh pony	M	Showing	2	N
6	TB	G	Eventing	2	N
11	TB	G	General	3	N
6	Irish sports horse	G	Eventing	2	N
11	Cob	G	General	3	N
9	Irish Sports Horse	G	General	2	N
13	Welsh D	G	General	2	N
9	Irish Sports Horse	M	Eventing	2	N
10	Warmblood	M	Showjumping	3	Y
13	New Forest	G	General	3	Y
14	Warmblood	G	General	2	N
5	Warmblood	G	Dressage	2	N
10	Thoroughbred	G	General	2	N
8	Pony cob	G	General	2	N
12	Warmblood	G	Dressage	3	Y

*Note*: Headshaking grade was scored using a three‐point scale as described by Roberts et al.^15^

Abbreviations: G, gelding; M, mare; Y, years.

**TABLE 2:** Signalment and headshaking (HS) details of non‐trigeminal mediated headshaking horses (*n* = 10).

Age (y)	Breed	Sex	Use	Grade/3	Self‐mutilation	Diagnosis
10	Connemara	M	General	2	N	Multi‐limb lame
6	Warmblood	M	Dressage	3	N	Periodontal disease
4	Warmblood	M	Dressage	3	N	Neck OCD
14	Irish Sports Horse	G	General	3	N	Cervical facet arthropathy
5	Cob	M	General	2	N	TMJ pathology (responded to medication)
4	Cob	M	General	2	N	Neck pain
4	Cob	M	General	2	N	Back pain
20	Cob	G	General	3	N	TMJ pathology (responded to medication)
16	Welsh	G	General	2	N	TMJ pathology (responded to medication)
10	Thoroughbred Cross	G	General	2	N	Sinusitis

*Note*: Headshaking grade was scored using a three point scale as described by Roberts et al.^15^

Abbreviations: G, gelding; M, mare; OCD, osteochondrosis dissecans; TMJ, temporomandibular joint; Y, years.

No significant differences were found across the four horse groups for all accelerometer data with the exception of mean positive g (*P* = 0.01) (Table 3). Post‐hoc analysis identified that TGMHS horses recorded increased mean positive g compared with CONTROLS (*P* = 0.014).

**TABLE 3:** Median and interquartile range (IQR) accelerometer vertical (*Z* axis) data for control (*n* = 16), lame (*n* = 12), non‐trigeminal mediated headshaking (non‐TGMHS) (*n* = 10) and trigeminal mediated headshaking (TGMHS) (*n* = 18; highlighted row) horses.

Group	Value	Ratio *n* +VE peaks >1 g:*n* −VE peaks <1 g	Mean +VE (g)	Mean –VE (g)	Max +VE (g)	Min –VE (g)	%>+2 g	%<−2 g	% −2 g and +2 g	Peaks>1 g or <1 g/min	+VE peaks >1 g/min	−VE peaks <1 g /min
Controls	Median	0.7	1.3	−1.2	2.5	−2.3	0.6	0.1	0.8	127.4	43.6	91.5
	IQR	0.8	0.1	0.1	1.0	1.1	0.8	0.8	0.8	179.4	67.4	94.0
Lame	Median	0.4	1.3	−1.3	2.7	−2.2	1.6	0.3	3.5	102.2	37.3	66.8
	IQR	0.6	0.5	0.2	1.4	1.1	4.1	2.7	5.8	115.7	54.3	66.2
Non‐TGMHS	Median	0.2	1.4	−1.3	3.9	−3.6	1.7	3.6	6.8	77.8	20.3	60.7
	IQR	0.2	0.4	0.3	1.4	0.8	3.8	3.9	8.5	58.9	16.9	38.5
All except TGMHS	Median	0.3	1.4	−1.3	2.8	−2.7	0.7	0.9	2.6	82.5	26.3	62.7
	IQR	0.9	0.3	0.2	1.8	1.6	1.8	3.0	6.1	110.4	41.3	79.2
TGMHS	Median	1.0	1.7	−1.5	4.2	−3.8	9.2	3.9	13.9	127.6	46.5	66.0
	IQR	0.9	0.3	0.4	2.2	3.1	8.1	9.7	17.8	75.4	33.6	56.2

*Note*: No significant differences were found across the four horse groups for all accelerometer data with the exception of mean positive g (*P* = 0.01). Post hoc analysis identified that TGMHS horses recorded increased mean positive g compared with CONTROLS (*P* = 0.014).

Abbreviations: %, percentage; +VE, positive; −VE, negative; g, gravity; Max, maximum; Min, minimum; peaks/min, peaks per minute.

**FIGURE 3:**
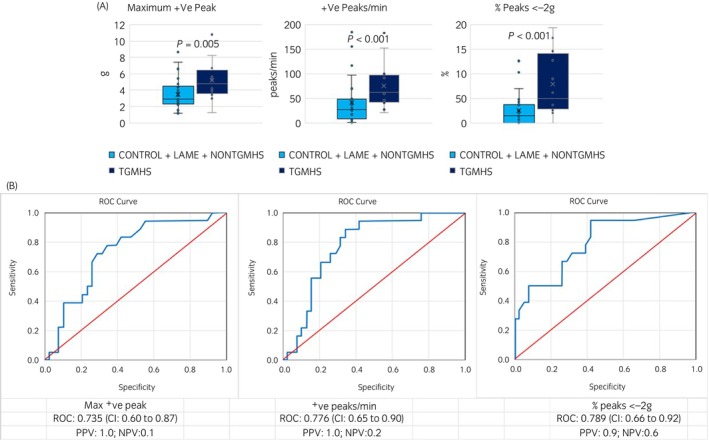
(A) Vertical (*Z* axis) accelerometer data variables with acceptable discrimination between trigeminal‐mediated headshaking horses (TGMHS) and CONTROL, LAME and non‐trigeminal‐mediated headshaking (NONTGMHS) horses combined. (B) Receiver operator characteristic (ROC) curve values and confidence intervals (CI) for vertical (*Z* axis) accelerometer data. +VE, positive; −VE, negative; NPV, negative predictive value; PPV, positive predictive value.

**FIGURE 4:**
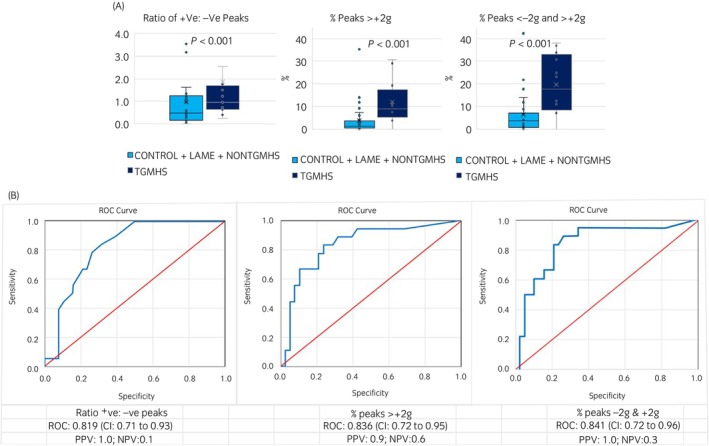
(A) Vertical (*Z* axis) accelerometer data variables with excellent discrimination between trigeminal‐mediated headshaking horses (TGMHS) and CONTROL, LAME and non‐trigeminal‐mediated headshaking (NONTGMHS) horses combined. (B) Receiver operator characteristic (ROC) curve values and confidence intervals (CI) for vertical (Z axis) accelerometer data. +VE, positive; −VE, negative; NPV, negative predictive value; PPV, positive predictive value.

Median and interquartile accelerometer data are shown in Table 3. Compared with all other horses combined, TGMHS horses had a significantly greater ratio of positive to negative peaks (3.5 times higher, *P* < 0.001), mean and maximum positive g (1.3 times higher, *P* = 0.005; 1.5 times higher, *P* = 0.005), percentage of peaks >+2 g (12.1 times higher, *P* < 0.001), mean and minimum negative g (both 1.5 times lower, *P* < 0.001), percentage of peaks <−2 g (4.5 times higher, *P* < 0.001), percentage of peaks <−2 g and >+2 g (5.6 times higher, *P* < 0.001) and number of positive peaks per minute (2.4 times higher, *P* < 0.001) (Figures 3A and 4A).

ROC AUC and respective confidence intervals accompanied by threshold values for individual accelerometer variables to discriminate a horse having TGMHS are shown in Table 4. Accelerometer variables with acceptable ROC area under curve (AUC) values (0.7–0.8) for discrimination were mean and maximum positive g (CI 0.59–0.88; 0.60–0.87), positive peaks per minute (CI 0.65–0.90) and percentage of peaks <−2 g (CI 0.66–0.92). Values showing excellent discrimination (0.8–0.9) were ratio of positive to negative peaks (CI 0.71–0.93), percentage of peaks >+2 g (CI 0.72–0.95) and percentage of peaks <−2 g and >+2 g (CI 0.72–0.96) (Figures 3B and 4B). PPV for all measures are high (≥0.9) suggesting the proposed thresholds discriminate well for true positive cases, however the low NPV also indicate the thresholds cannot differentiate a false negative case well.

**TABLE 4:** Receiver operator characteristics (ROC) area under the curve (AUC), confidence intervals (CI), threshold values, specificity and sensitivity values for accelerometer variables to discriminate a horse having TGMHS.

Measure	ROC AUC	CI	Threshold value	Sensitivity	Specificity
Ratio *n* +VE peaks >1 g:*n* −VE peaks <1 g	0.82	0.71–0.93	0.05	1.00	0.87
Mean +VE (g) peak	0.74	0.59–0.88	1.15	0.94	0.90
Mean −VE (g) peak	0.18	0.06–0.33	−1.85	0.81	0.96
Max +VE (g) peak	0.74	0.60–0.87	1.65	0.95	0.84
Min −VE (g) peak	0.20	0.09–0.40	−6.7	0.83	1.00
% Peaks >+2 g	0.84	0.72–0.95	−1.00	1.00	1.00
% Peaks <−2 g	0.79	0.66–0.92	−1.00	1.00	1.00
% Peaks <−2 g and >+2 g	0.84	0.72–0.96	0.15	0.94	0.82
Peaks >1 g or <1 g/min	0.60	0.36–0.73	41.70	0.88	0.83
+VE peaks >1 g/min	0.78	0.65–0.90	5.10	1.00	0.82
−VE peaks <1 g/min	0.46	0.28–0.64	15.50	0.88	0.83

Abbreviations: %, percentage; +VE, positive; −VE, negative; g, gravity; Max, maximum; Min, minimum; peaks/min, peaks per minute.

## 4 DISCUSSION

This is the first report of triaxial accelerometer use for measurement of headshaking in horses. The accelerometer was able to provide quantitative data of the characteristics and frequency of headshaking movements. Additionally, data analysis provided excellent discrimination of headshaking‐associated movements of the head from normal head motion displayed by control and FL horses, and between TGMHS and non‐TGMHS. As such, the accelerometer holds potential as a diagnostic aid to the clinician, as well as being useful in objective quantitation and monitoring, of disease severity.

Characteristics of head movements differed between groups of horses. TGMHS horses had both more frequent head movements and movements with greater g‐force. TGMHS horses had over twice as many positive peaks per min than other horses and the percentage of peaks >+2 g was over 12 times greater and the percentage of peaks <−2 g was over 4 times greater than for other horses. These rapid acceleration/deceleration peaks are associated with the head movement described by owners as when the horse looks like it has received a sudden electric shock to the muzzle or been stung by a bee up its nose.

Positive and negative predictive values were very good at predicting a TGMHS but not good generally at identifying a false negative case creating a lot of false positives in the group ‘all except TGMHS’. An ROC >0.80 is the minimum acceptable limit for results of clinical significance and, whilst this initial study shows the use of accelerometers is promising, further work is warranted to optimise their clinical utility. Horses with TGMHS have a 10‐fold reduction in activation threshold of the trigeminal nerve.^2^ As such, the violent head movements seen in TGMHS are interpreted as indicative of trigeminal neuropathic pain, which is described by patients with human trigeminal neuralgia as sudden, lancinating, itching, tingling, burning and electric shock‐like sensations.^21^ Severely affected horses may headshake even at rest and be so distressed that humane euthanasia is warranted on welfare grounds, as well as being unsafe to handle.^1^ If frequency of violent head movements is taken as a proxy for disease severity, quantitative data collection with an accelerometer allows more objective interpretation of compromised welfare.

The absence of a gold standard diagnostic test for TGMS necessitates a diagnosis by exclusion of other causes by detailed physical examination, endoscopy and CT. Although a decreased activation threshold of the trigeminal nerve has been reported in headshaking horses compared with controls such electrophysiologic measurements must be performed under general anaesthesia and are therefore not justified in clinical cases.^2^ Additionally, to date, electrophysiological data from TGMHS and non‐TGMHS horses have not been compared. Careful observation by experienced clinicians can usually distinguish headshaking from normal head movements seen in healthy or those associated with lameness, but it can be impossible to discriminate between TGMHS and non‐TGMHS on observation alone. The advent of CT has shown that 10% of horses presented for headshaking that undergo CT have non‐TGMHS.^3^ Cost of CT precludes some owners undertaking advanced imaging studies and therefore use of an accelerometer could provide useful additional data to increase index of suspicion of TGMHS, at minimal cost. The reason for an increased proportion of non‐TGMHS horses in this population (36%) is unclear but may reflect the complex referral caseload that the authors see.

In this study, data were only collected over a 5 min exercise period, however longer periods of data collection are possible with the device used (up to 7 days), which could be useful in monitoring horses with very intermittent headshaking and also in identifying specific, variable triggers, for example, relating to the environment or weather. As such, accelerometer use could be useful to both veterinary surgeons and owners of headshaking horses. Research with the accelerometer involving machine learning may add further benefit to its use.

A limitation of the study is the relatively low numbers of horses in each group however the narrow range of confidence intervals reported support rejection of the null hypothesis.^22^ Additionally, control horses were not evaluated specifically for lameness by an orthopaedic surgeon however, as these horses presented for prepurchase examination, it is reasonable to expect that lameness would have been identified. TMHS horses did not undergo specific gait and vertebral column evaluation as this is not part of a routine headshaking investigation. Finally, only *Z* axis data were analysed in this study due to the predominance of vertical head motion but analysis of data from other axes may also prove useful.

In conclusion, a tri‐axial accelerometer mounted at the poll provides accurate quantitation of headshaking and demonstrated excellent discrimination of TGMHS horses from non‐TGMHS, control and lame horses.

## FUNDING INFORMATION

The Langford Trust for Animal Health and Welfare.

## CONFLICT OF INTEREST STATEMENT

The authors declare no conflicts of interest.

## AUTHOR CONTRIBUTIONS

**Kirstie Jane Pickles:** Conceptualization; investigation; writing – original draft; methodology; validation; project administration. **David J. Marlin:** Writing – review and editing; software; data curation; methodology; validation. **Jane Michelle Williams:** Writing – review and editing; data curation; formal analysis. **Veronica L. H. Roberts:** Investigation; funding acquisition; methodology; validation; writing – review and editing; project administration.

## DATA INTEGRITY STATEMENT

All authors have had full access to all data in the study and take responsibility for the integrity of the data and accuracy of data analysis.

## ETHICAL ANIMAL RESEARCH

The study received University of Bristol ethics approval, registered with Veterinary Investigation Number 20027 and University of Nottingham ethical review approval number 3426‐210‐921.

## INFORMED CONSENT

Informed consent was obtained from horse owners.

### PEER REVIEW

The peer review history for this article is available at https://www.webofscience.com/api/gateway/wos/peer-review/10.1111/evj.14132.

## ANTIMICROBIAL STEWARDSHIP POLICY

Not applicable.

## Supporting information


**Table S1.** Receiver operator characteristics (ROC), area under the curve (AUC) and confidence interval (CI) values for accelerometer variables to discriminate a horse having TGMHS from horses with NON‐TGMHS.

## Data Availability

The data that support the findings of this study are available from the corresponding author upon reasonable request: Open sharing exemption granted by editor.
