# Supplementary material for: Use of a poll‐mounted accelerometer for quantification and characterisation of equine trigeminal‐mediated headshaking
Source: Equine Vet J. 2024 Jul 17;57(3):645–53. doi: 10.1111/evj.14132 (PMC11982430; doi:10.1111/evj.14132)
Supplement: Supplementary file 1 — Table S1. Receiver operator characteristics (ROC), area under the curve (AUC) and confidence interval (CI) values for accelerometer variables to discriminate a horse having TGMHS from horses with NON‐TGMHS. [file EVJ-57-645-s001.pdf]

Equine Veterinary Journal BEVA

Table S1: Receiver operator characteristics (ROC), area under the curve (AUC), and confidence interval (CI) values for accelerometer variables to discriminate a horse having TGMHS from horses with NON-TGMHS.

Max = maximum; Min = minimum; +VE = positive; -VE = negative; g = gravity; peaks/min = peaks per minute; % = percentage.

**Measure**	**ROC AUC**	**CI**	
Ratio n +VE peaks >1g:n -VE Peaks <1g	.83	.59	1.0
Mean +VE (g) Peak	.61	.32	.91
Mean -VE (g) Peak	.32	.05	.59
Max +VE (g) Peak	.57	.31	.78
Min -VE (g) Peak	.42	.18	.66
% Peaks >+2g	.71	.44	.97
% Peaks <-2g	.64	.40	.89
% Peaks <-2g & >+2g	.67	.41	.93
Peaks >1g or <1g/min	.65	.42	.88
+VE peaks >1g/min	.86	.72	1.0
-VE peaks <1g/min	.50	.26	.75
